# Combined Treatment with Fasudil and Menthol Improves Functional Recovery in Rat Spinal Cord Injury Model

**DOI:** 10.3390/biomedicines8080258

**Published:** 2020-07-31

**Authors:** JeongHoon Kim, Hari Prasad Joshi, Kyoung-Tae Kim, Yi Young Kim, Keundong Yeo, Hyemin Choi, Ye Won Kim, Un-Yong Choi, Hemant Kumar, Seil Sohn, Dong Ah Shin, In-Bo Han

**Affiliations:** 1 Department of Neurosurgery, CHA University School of Medicine, CHA Bundang Medical Center, Seongnam-si 13496, Korea; jhkim16@kakao.com (J.K.); hariprasadjoshi10@gmail.com (H.P.J.); irenekim1102@gmail.com (Y.Y.K.); keundong182@gmail.com (K.Y.); littlechoi88@gmail.com (H.C.); shn01043@naver.com (Y.W.K.); nschoiuy@gmail.com (U.-Y.C.); sisohn@cha.ac.kr (S.S.); 2 Department of Neurosurgery, School of Medicine, Kyungpook National University, Daegu 41944, Korea; nskimkt7@gmail.com; 3 Department of Neurosurgery, Kyungpook National University Hospital, Daegu 41944, Korea; 4 Department of Pharmacology and Toxicology, National Institute of Pharmaceutical Education and Research (NIPER)-Ahmedabad, Gandhinagar, Gujarat 382355, India; hemantbhave@gmail.com; 5 Department of Neurosurgery, Yonsei University College of Medicine, 50 Yonsei-ro, Seodaemun-gu, Seoul 03722, Korea

**Keywords:** fasudil, menthol, spinal cord injury, neuroprotection, neurite regeneration

## Abstract

Neuroprotective measures by preventing secondary spinal cord injury (SCI) are one of the main strategies for repairing an injured spinal cord. Fasudil and menthol may be potent neuroprotective agents, which act by inhibiting a rho-associated protein kinase (ROCK) and suppressing the inflammatory response, respectively. We hypothesized that combined treatment of fasudil and menthol could improve functional recovery by decreasing inflammation, apoptosis, and glial scar formation. We tested our hypothesis by administering fasudil and menthol intraperitoneally (i.p.) to female Sprague Dawley rats after moderate static compression (35 g of impounder for 5 min) of T10 spinal cord. The rats were randomly divided into five experimental groups: (i) sham animals received laminectomy alone, (ii) injured (SCI) and untreated (saline 0.2 mL/day, i.p.) rats, (iii) injured (SCI) rats treated with fasudil (10 mg/kg/day, i.p.) for two weeks, (iv) injured (SCI) rats treated with menthol (10 mg/kg/day, i.p.) for twoweeks, (v) injured (SCI) rats treated with fasudil (5 mg/kg/day, i.p.) and menthol (10 mg/kg/day, i.p.) for two weeks. Compared to single treatment groups, combined treatment of fasudil and menthol demonstrated significant functional recovery and pain amelioration, which, thereby, significantly reduced inflammation, apoptosis, and glial/fibrotic scar formation. Therefore, combined treatment of fasudil and menthol may provide effective amelioration of spinal cord dysfunction by a synergistic effect of fasudil and menthol.

## 1. Introduction

Traumatic spinal cord injury (SCI) is a devastating condition with far-reaching physical, emotional, and economic consequences for patients, families, and society at large [1]. Traumatic SCI represents a heterogeneous and complex pathophysiology [1].Major efforts have been made to develop a variety of pharmacological and stem cell-based therapies for SCI. However, a universally efficacious treatment for SCI in humans remains elusive. The number of neuroprotective strategies ranging from surgical decompression to potential pharmacological agents as methylprednisolone [2], minocycline [3], riluzole [4], granulocyte colony stimulating factor (G-CSF) [5], and therapeutic hypothermia (32 °C–34 °C) [6] have been applied and clinical trials are undergoing. Unfortunately, none of them have been approved as the sole treatment strategy for SCI.

Recent studies reported that the ras homolog family member A (RhoA) pathway plays a critical role in the pathophysiology of SCI [7,8]. After SCI, myelin inhibitors such as nogo-A promote Rho activation and downstream activation of rho-associated kinase (ROCK), which leads to growth cone collapse and to axonal growth arrest at the lesion site [9,10]. Activated ROCK phosphorylates various downstream markers, including phosphatase and tensin homologue (PTEN), myosin light chain (MLC), and glial fibrillary acidic protein (GFAP), which regulate cellular responses such as the induction of apoptosis, stress fiber formation, and neurite retraction [11]. Therefore, inhibiting RhoA-ROCK signaling pathway has emerged as a promising approach to repair the injured spinal cord [10]. According to research on the activation of ROCK, due to ROCK’s carboxyl terminal, its kinase activity is auto-inhibited and ROCK is enzymatically inactive in its native form [12]. ROCK is activated either by disrupting the auto-inhibitory region with active Rho-GTP (Rho-dependent activation), which was promoted after SCI or by removing the carboxyl terminal with caspase-3 or granzyme B (Rho-independent activation).

Rho inhibition mediates axonal regrowth and neuroprotection, which leads to functional recovery after SCI. For example, cethrin (VX-210), the recombinant deactivator of RhoA with dura and cell membrane penetrance, has shown significant neurological improvement in patients with SCI [8,13]. While there have been many attempts to apply RhoA-ROCK pathway inhibitors in the treatment of SCI, after comparing the treatment effects of fasudil, Y-27632, and C3 exoenzyme (a RhoA inhibitor), fasudil was identified as the most efficient at improving functional recovery after SCI in a rat model [14,15]. The in-vitro experiment also represents that murine neuroblastoma Neuro2a (N2a) cells showed dose-dependent neurite outgrowth when treated with fasudil [16]. The wide range of ROCK inhibitors including fasudil, Y-27632, ripasudil, hydroxyfasudil, netarsudil, H-1152, KD-025, and AMA-0076 are available. Currently, only fasudil (HA-1077), its derivative ripasudil (K-115), and netarsudil (AR-13324) have been licensed for clinical use. However, many other ROCK inhibitors are undergoing clinical trials [9].

Yet, before fasudil can be implemented clinically, there are practical problems to be solved. First, delayed treatment with a Rho-kinase inhibitor does not promote functional recovery in a rat SCI model [17]. While immediate treatment with fasudil enhances locomotor activity after SCI, fasudil has a limited pharmacological efficacy in cases of chronic SCI, where glial scar tissue is already established [14,17]. In addition, previous studies have shown that upregulating inflammatory pathways, especially the cyclooxygenase-2 (COX-2) pathway, could cause resistance of ROCK inhibitors [18], which suggests that the therapeutic effect of ROCK inhibitors would be enhanced when combined with COX-2 inhibitors, such as celecoxib. Determining the optimal dose of the ROCK inhibitor to use is an important issue. While ROCK inhibitors have dose-dependent beneficial effects on axon sprouting, low doses of ROCK inhibitors do not enhance axon sprouting or detrimental effects inin vivo [19]. A clinical trial has also revealed that fasudil may lead to some adverse effects including intracranial bleeding, hypotension, hepatic and hepatobiliary disorders, and reversible renal dysfunction [20,21]. Therefore, fasudil’s limitations include a low potency, lack of selectivity, and adverse effects. These limitations have encouraged the development of additional novel, specific, and potent therapeutic strategies for neuronal regeneration after SCI.

Menthol, on the other hand, whichis a natural compound found in mint leaves and many essential natural oils, reduces glutaminergic neurotoxicity, decreases inflammation, and suppresses COX-2 expression in mice [14], and, thus, exertsa neuroprotective and analgesic effect [21]. It suppresses the inflammatory processes and enhances antioxidant activity to exert therapeutic effects on various diseases [22]. Menthol is also a specific agonist of transient receptor potential melastatinsubtype 8 (TRPM8) and, in low concentrations, it alleviates mechanical allodynia and cold hyperalgesia [23]. Menthol’s central therapeutic activity is correlated with blockage of voltage-gated sodium and calcium channels [24]. Menthol has been reported for its anti-neuroinflammatory effects on microglial activation in mice [25]. Based on the previously reported neuroprotective impacts of these substances, in the present study, we hypothesized that combined treatment of fasudil and menthol could promote motor function recovery and pain amelioration following SCI in a rat model. Furthermore, we believethat combined synergistic effects could be themain basis for their therapeutic effects. We also examined whether the combined treatment improved functional recovery after SCI by alleviating apoptosis, inflammation, and glial/fibrotic scar formation, and by promoting angiogenesis and neuroprotection in a rat model of SCI.

Wetested the hypothesis that the combination of fasudil and menthol may be superior to the use of either treatment alone, and we believed that significant functional and pain recovery along with attenuation of inflammation, apoptosis, and glial scar formation, following combined fasudil and menthol treatment, could be due to their synergistic effects.

## 2. Materials and Methods

### 2.1. Drugs

**Figure 1:**
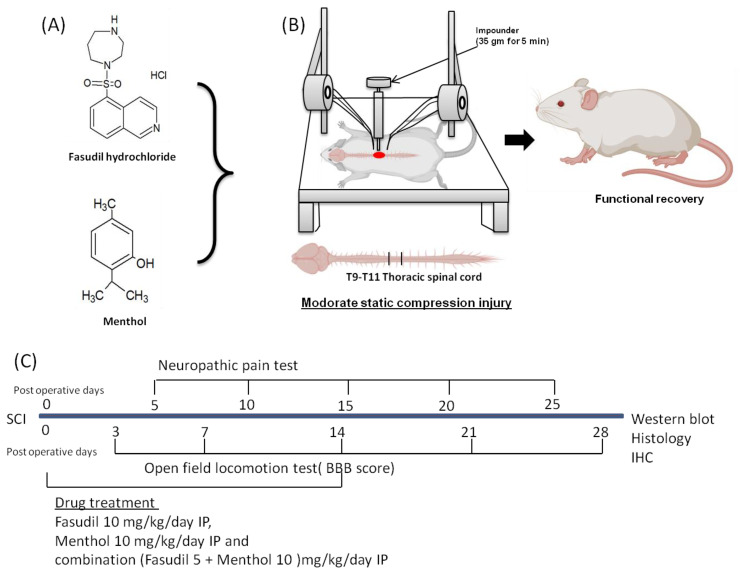
Description of SCI model rat experiment. (**A**) chemical structures of fasudil hydrochloride and menthol. (**B**) After moderate static compression spinal cord injury (SCI) on T10, functional recovery was evaluated. (**C**) Experimental design of behavior analysis, terminal deoxynucleotidyl transferase dUTP nick end labeling (TUNEL) assay, and immunohistochemistry (IHC). i.p., intraperitoneally.

Fasudil hydrochloride C14H17N3O2S.HCL (291.36 g/mol) and (-)-Menthol; C10H20O, 156.269 g/mol) (levo isomer) were purchased from Sellechem, Houston, USA. Both the drugs were obtained in lyophilized form and reconstituted in dimethylsulphoxide before administration (Figure 1A).

### 2.2. Neurite Outgrowth and Cell Viability Assessment

In order to compare the neurite outgrowth and cell viability, we used mouse N2a cells derived from a murine neuroblastoma. N2a cells were cultured in DMEM with high glucose and with 10% fetal bovine serum (FBS), 100 unit/mL penicillin at 37 °C, 5% CO_2_ in a humidified incubator. We dispensed 100 µL of cell suspension (1 × 104 cells/well) in 96-well plates. After 24 h of incubation, the medium was replaced with the culture medium containing fasudil and menthol under various experimental conditions (control group (normal saline), lipopolysaccharide (LPS) 1 µg/mL group, LPS in combination with fasudil 250 µmol/L group, LPS with menthol 250 µmol/L group, LPS with fasudil 250 µmol/L and menthol 250 µmol/L, and LPS with fasudil 250 µmol/L and menthol 500 µmol/L group). Cells were documented via light microscope for 1 h and for 4 h after drug treatment. N2a cells neurite outgrowth was estimated by calculating the percentage of cells with neurites. After 24 h of incubation, cell viabilities were measured with the CCK-8 kit (Dojindo laboratories, kumamoto, Japan). We added 10 µL of CCK-8 solution to each well of the plates and incubated the plate for an additional 3 h in the incubator. Optical density (O.D.) at 450 nm was measured with a microplate reader.

### 2.3. Animal Model and Group

Total 46 young adult (7-week aged) female Sprague-Dawley (SD) rats (body weight: 200–220 g) used in this study were purchased from Orient Bio Inc (Seongnam, Korea). Animals were housed in a facility at 55–65% humidity with a controlled temperature of 24 ± 3 °C and light/dark cycles of 12 h.They had free access to food and water. All animal procedures were performed according to the approved protocol by the Institutional Animal Care and Use Committee (IACUC) of CHA University (IACUC180089, approved on 18 April 2018).

After 1 week of the adaptation period under experimental condition, the surgical operation was started by anesthesia with the mixture of Zoletil^®^ (50 mg/kg, i.p. Virbac Laboratories, Carros, France) and Rompun^®^ (10 mg/kg, i.p. Bayer, Seoul, Korea) via the intraperitoneal route. Complete anesthesia was assessed using hindlimb withdrawal in response to a noxious foot pinch. After skin preparation and precise positioning of anesthetized rats, a laminectomy was performed to expose the T10 spinal cord [26]. The exposed dorsal surface of the spinal cord was subjected to moderate, static-weight compression injury (35 g/5 min) [27] (Figure 1B). Following compression injury, the surgical site was closed by suturing the muscle, fascia, and skin, which is followed by external povidone-iodine application. Animals were placed on a heating pad to maintain body temperature and 0.9% normal saline (5 mL) was injected by a subcutaneous route. Rats were given a prophylactic treatment following SCI by injecting antibiotic (cefazolin, CKD Pharmaceuticals, Seoul, South Korea) and analgesic factors (ketoprofen, SCD Pharm. Co. Ltd., Seoul, South Korea).Then 5 mL of 0.9% sterile saline was injected subcutaneously. Manual bladder expression of urine was performed twice daily until a bladder reflex was established.

Prior to surgery, rats were randomly divided into five experimental groups consisting of: a sham group (those underwent a laminectomy at the T10 level without compression injury or receiving any pharmacological treatments, *n* = 6), injury-alone group (rats underwent a weight compression injury (SCI), which was followed by normal saline (ns) injection, 0.2 mL/day i.p., *n* = 10), fasudil-treated group (SCI animals treated with fasudil 10 mg/kg/day, i.p., *n* = 10), menthol-treated group (SCI animals treated with menthol 10 mg/kg/day, i.p., *n* = 10), and a combination group (SCI animals treated with fasudilof 5 mg/kg/day in combination with menthol of 10 mg/kg/day, *n* = 10). Intraperitoneal administration of fasudil and menthol was conducted once every day for 2 weeks. Rats were sacrificed for histological examination 1 day after surgery and 4 weeks after surgery (Figure 1C).

### 2.4. Behavioral Analysis

#### 2.4.1. Hind Limb Locomotor Score (Basso, Beattie, and Bresnahan (BBB) Score)

Hindlimb motor function was evaluated using the open-field Basso, Baettie, and Bresnahn (BBB) locomotor test 3, 7, 14, 21, and 28 days following injury as per the previously reported method [28]. Briefly, BBB comprised of a 22-point scale (with scores of 0–21) that systematically and logically determines the hind limb recovery from complete paralysis (score of 0) to a complete recovery (score of 21). Therefore, an increase in score represents the gradual recovery of the hind limb. It was evaluated by allowing animals to walk freely on the open-field surface for at least 3 min. The animals’ hindlimb locomotor score was evaluated by two experienced investigators who were blinded to the experimental plan and condition.

#### 2.4.2. Test for Neuropathic Pain

Neuropathic pain was checked using Von Frey filaments (Bioseb) as per the reported method [29]. Animals were acclimatized in rat chambers (Ugo Basile) and filament probing was done when the animals were calm and not moving. The simplified up-down method (started with 2 g) was used to determine the paw withdrawal threshold (PWT)with Von Frey filaments before surgical operation (day 0, data not shown) and 5, 10, 15, 20, and 25 days following injury to minimize interference between behavioral experiments.

A positive response included flinching, licking, vocalization, or overt behavioral cues corresponding to discomfort. A total of 5 stimuli per test were recorded and the average of 3 readings (lowest and highest removed) was used to determine the average PWT after injury or treatment. The average PWT was evaluated by two experienced investigators who were blinded to the experimental design.

### 2.5. TUNEL Assay

The assay was performed by the protocol provided by the TUNEL assay kit (TACS^®^2 TdT-DAB in Situ Apoptosis Detection kit). Spinal cords were collected 1 day after SCI and were preserved in 4% paraformaldehyde (PFA) for 24 h. The following day, tissues were processed and embedded in paraffin. Sections between 6–10 µm were collected for the assay. Histologic sections were deparaffinized, dehydrated, and washed in 1 × PBS for 10 min. Sections were then incubated with proteinase k solution for 1 h, which was followed by quenching. Sections were then labeled by a 1 × TdT labeling buffer, and the TUNEL reaction mixture (Labeling Reaction Mix) and the reaction were stopped by a stop buffer. In the next step, Streptavidin-HRP was added to each section and slides were then immersed in a 3, 3-Diaminobenzidine (DAB) solution and counterstained with 1 percent methyl green for 5 min. Sections were then washed, dehydrated, and cleared, which was followed by mounting. The numbers of apoptotic bodies were examined by OLYMPUS (U-TVO.63XC, Tokyo, Japan).

### 2.6. Immunohistochemistry (IHC)

On day 3 and 28 following SCI, animals were anesthetized with a mixture of Zoletil^®^ (50 mg/kg, Virbac Laboratories, Carros, France)/Rompun^®^ (10 mg/kg, Bayer, Seoul, Korea) solution i.p. Rats were transcardially perfused first with 4% PFA and then with 0.9% saline. A 10-mm section of the spinal cord centered at the lesion site was dissected out and post-fixed in the same fixative (4% PFA) overnight. The tissues were dehydrated through a gradient of ethyl alcohol solutions. PFA was removed by keeping tissue in xylene overnight. Spinal cords were embedded in paraffin and cut into serial longitudinal sections with a thickness of 5 to10 μm. Tissue sections were selected, dewaxed, and stained with antibodies against Angiopoietin-1 (ANGPT-1, 1:200, Abcam, Cambridge, UK), Neurofilament (NF, 1:1000, Abcam), Brain-derived neurotrophic factor (BDNF, 1:300, Almone Lab), Glial fibrillary acidic protein (GFAP, 1:500, Abcam), Ionized calcium-binding adapter molecule -1 (IBA-1, 1:200, WAKO chemicals, USA), Caspase-3 (1:50, Abcam), CD68 (1:200, Abcam), transforming growth factor-β1 (TGF-β1, 1:100, Abcam), Arginase-1 (Arg-1, 1:100, Abcam), Growth-associated protein-43 (GAP-43, 1:500, Abcam), growth cone antibody (1:200, Abcam), Anti-Collagen-IV (1:500, Abcam), and Anti-Connexin-43 (1:500, Abcam). After 24 h, sections were washed with PBS-T (Phosphate-Buffered Saline- tween 20) and incubated with secondary antibodies including donkey anti-rabbit Alexa Fluor^TM^ 647 (Invitrogen), goat anti-rabbit Alexa Fluor^TM^ 488 (Invitrogen), donkey anti-mouse Alexa Fluor^TM^ 488 (Invitrogen), donkey anti-rabbit Alexa Fluor^TM^ 568 (Invitrogen), and chicken anti-goat Alexa Fluor^TM^ 647 (Invitrogen). Following secondary antibody incubation, sections were stained with 4′,6-diamidino-2-phenylindole (DAPI, 1:500) for 10 min. Sections were mounted and examined using a fluorescence microscope (Zeiss 880, Oberkochen, Germany and Leica SP5, Mannheim, Germany).

### 2.7. Luxol Fast Blue (LFB) Staining

To determine the rate of myelin preservation following drug treatment, LFB staining was performed and lesion volume was measured. With spinal cord sections having a distinct injury epicenter (0 mm), rostral (1 mm), and caudal (2 mm) segments were selected. Histologic sections were deparaffinized, dehydrated in graded alcohol solutions, and incubated in an LFB (Luxol fast Blue) solution at room temperature for 24 h. Sections were then differentiated in a lithium carbonate solution, which is followed by 70% ethyl alcohol, and counterstained with cresyl violet. Slides were then dehydrated, cleared, and mounted by a mounting solution. Lastly, slides were scanned with an OLYMPUS C-mount camera adapter (U-TVO.63XC, Tokyo, Japan). The total area of cavitation was measured by ImageJ (Wayne Rasband, NIH, Bethesda, MD, USA).

### 2.8. Statistical Analysis

All data were analyzed using Graph Pad Prism ver. 8.02 (Graph Pad, Inc., La Jolla, CA, USA). Unless stated, data are expressed as mean  ±  standard error of the mean (SEM). The BBB scores and nociception data were analyzed by two-way ANOVA, which was followed by a Bonferroni test. Fluorescence intensity was quantified by ImageJ 1.52a (NIH, USA), and graphs were analyzed by one-way ANOVA. This was followed by Neuman Keul’s test. *p*-values < 0.05 were considered statistically significant.

## 3. Results

### 3.1. Effect of Combined Fasudil and Menthol on Neurite Outgrowth in Neuro2A (N2a) Cells

**Figure 2:**
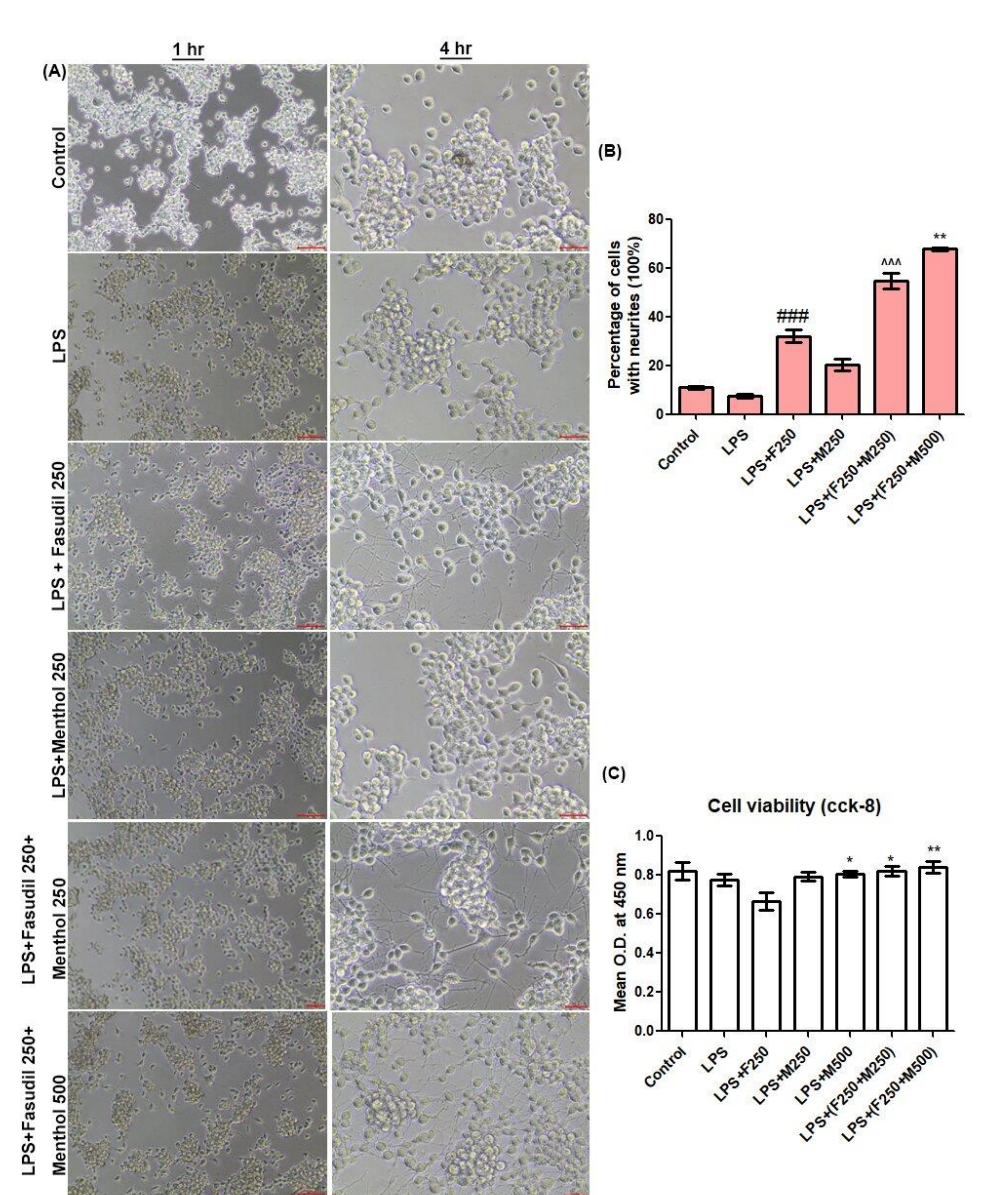
Effect of fasudil or menthol, alone or in combination on neuritis outgrowth in lipopolysaccharide (LPS)-stimulated murine Neuro2A (N2a) cells.(**A**) Representative N2a cells shown in optical microscope images at 1 h and 4 h after treatmentN2a cells treated with LPS showed dramatic inhibition of neurite outgrowth within 1 to 4 h after cell incubation. (scale bar = 200 µm) (**B**) Combined treatment of fasudil (250 µmol/L) and menthol (250 or 500 µmol/L) increased the percentage of cells with neuritis. All data are presented as the mean ± SEM of three independent experiments performed with *n* = 5. ### *p* < 0.001, vs. LPS, ^^^ *p* < 0.001, vs. LPS, ** *p* < 0.01, vs. LPS/fasudil 250 µmol/L treatment group. (**C**) O.D. at 450 nm estimated with Cell counting kit-8 assay revealed the effects of combined fasudil and menthol on mouse Neuro2A cell viability. The cell viability of the LPS combined with fasudil and menthol was relatively increased compared to individual fasudil or menthol-treated groups. Experiments were repeated three times. Data are presented as mean ±SEM. * *p* < 0.05, ** *p* < 0.01 vs. LPS/fasudil 250 µmol/L treated group. O.D., optical density. SEM, standard error of the mean.

After 24 h of pre-incubation, murine neuroblastoma N2a cells were treated with LPS, fasudil (250 µmol/L), and menthol (250 or 500 µmol/L). After 1 h and 4 h of incubation under experimental conditions, N2a cells treated with LPS showed dramatic inhibition of neurite outgrowth. Neurite outgrowth at 4 h was found to be significant than at 1 h, and a significant number of neurite outgrowth and longer neurite length was obtained in fasudil treatment groups compared to control cells, LPS-treated cells, and LPS/menthol (250 µmol/L) treated cells (Figure 2A) (### *p* < 0.001 vs. LPS). LPS/fasudil (250 µmol/L)/menthol (250 or 500 µmol/L) treatment group significantly promoted percentage of cells with neurite outgrowth even compared to the LPS/fasudil (250 µmol/L) treatment group (** *p* < 0.01 vs. LPS/Fasudil) (Figure 2B). After 24 h of incubation under experimental condition, CCK-8 assay was performed to evaluate cell viability and non-cytotoxicity of drug candidates at working concentrations after drug treatment. Mean O.D. at 450 nm was gradually decreased in the LPS group and LPS/fasudil-treated group when compared to the control group (Figure 2C). On the other hand, mean O.D (Optical density) at 450 nm was gradually increased in LPS/menthol treated group and LPS/fasudil/menthol (250 or 500 µmol/L) combination treatment group (* *p* < 0.05, ** *p* < 0.01 vs. LPS/fasudil), which displayed the optimal cell viability in a combined drug treatment group.

### 3.2. Effects of Fasudil or Menthol, Alone or in Combination, on Functional Recovery

**Figure 3:**
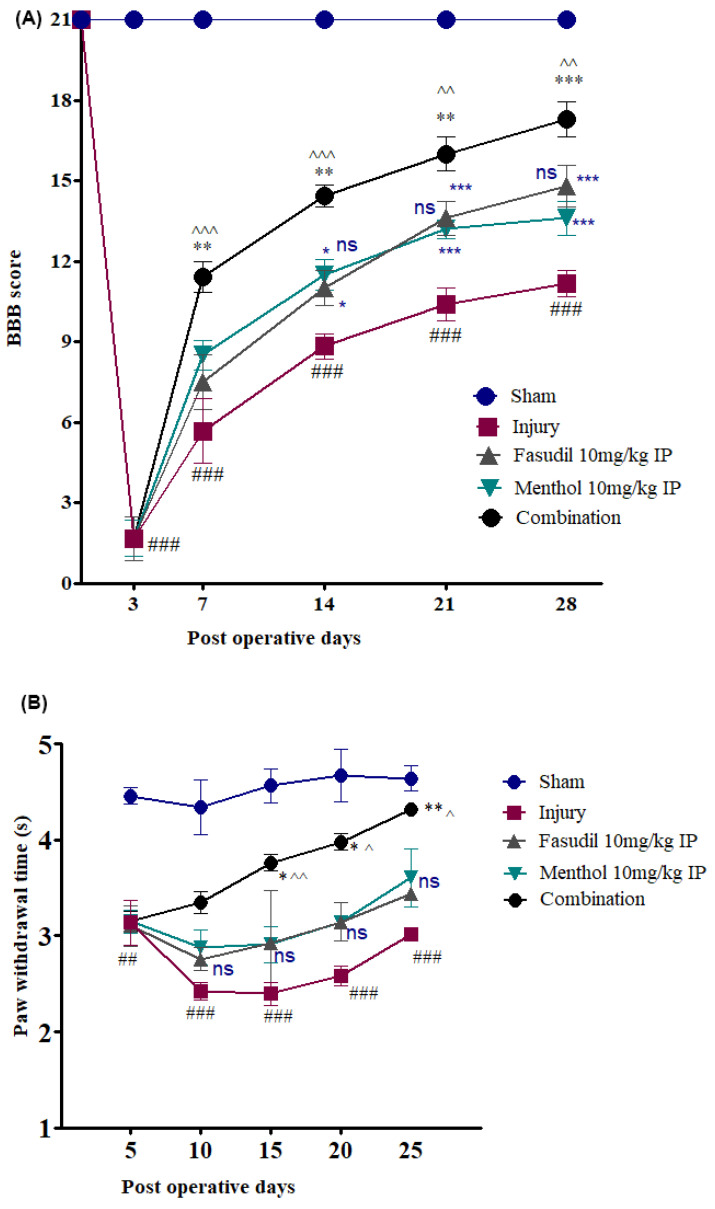
Combination of fasudil (5 mg) and menthol (10 mg) significantly improves functional recovery following spinal cord injury. Behavior analysis was performed from the day of surgery to 28 days after spinal cord injury (SCI). (**A**) Basso, Beattie, and Bresnahan (BBB) scores, (**B**) neuropathic pain test. Data are expressed as mean ± SEM and analyzed by two-way ANOVA followed by Bonferroni’s post-test. (*n* = 6/group). # *p* < 0.05, ## *p* < 0.01, ### *p* < 0.001 (sham vs. injury), ^ *p* < 0.05, ^^ *p* < 0.01, ^^^ *p* < 0.001 (combination vs. fasudil 10 mg), and * *p* < 0.05, ** *p* < 0.01, *** *p* < 0.001 (combination vs. menthol 10 mg).

All rats except sham group rats developed paraplegia, as reflected by their low BBB locomotor scores at threedays post-SCI. Modest functional improvements were observed in the injury alone group, which had a BBB score of 11.17 ± 0.48 (mean ± SEM) (Figure 3A) (### *p* < 0.001, sham vs. injury) at 28 days post-SCI. Moreover, gradually improved motor function was observed in single menthol and fasudil-treated rats with mean BBB scores of 14.8 ± 0.79 and 13.6 ± 0.6, respectively, at 28 days post-SCI. Combined treatment with menthol and fasudil led to a mean BBB score of 17.28 ± 0.65 (^^ *p* < 0.01, combination vs. fasudil) (*** *p* < 0.001, combination vs. menthol), (*** *p* < 0.001, combination vs. injury alone), and (*** *p* < 0.001, Sham vs. combination), which indicates a significantly enhanced locomotor function and faster recovery of effective motor coordination over that achieved with singular treatment with either drug alone (Figure 3A).

After SCI, neuropathic pain was evaluated with increased hypersensitivity (decrease in PWT) on days 5, 10, 15, 20, and 25. As evidence of considerable neuropathic pain, PWT in the injury-alone group was the lowest throughout the experiment, with a mean PWT of 3.01 ± 0.069 s at 25 days post-SCI (Figure 3B) (### *p* < 0.001, sham vs. injury). The fasudil group exhibited marginal improvements in the pain response at 25 days post-SCI, which was represented as a PWT of 3.44 ± 0.058 s (ns, fasudil vs. injury), (*** *p* < 0.001, Sham vs. fasudil) as did the menthol group (PWT of 3.60 ± 0.30 s) (ns, menthol vs. injury) (ns, menthol vs. fasudil) and (*** *p* < 0.001, sham vs. combination). The combination group exhibited significantly increased PWT (4.31 ± 0.027 s) (^ *p* < 0.05, ^^ *p* < 0.01, combination vs. fasudil) (* *p* < 0.05, ** *p* < 0.01, combination vs. menthol), (*** *p* < 0.001, combination vs. injury alone), and (ns, Sham vs. combination), which suggests that combined use of fasudil and menthol significantly alleviated neuropathic pain compared to singular treatment with either drug alone.

### 3.3. Effects of Fasudil or Menthol, Alone or in Combination, on Apoptosis and Inflammation Following SCI

**Figure 4:**
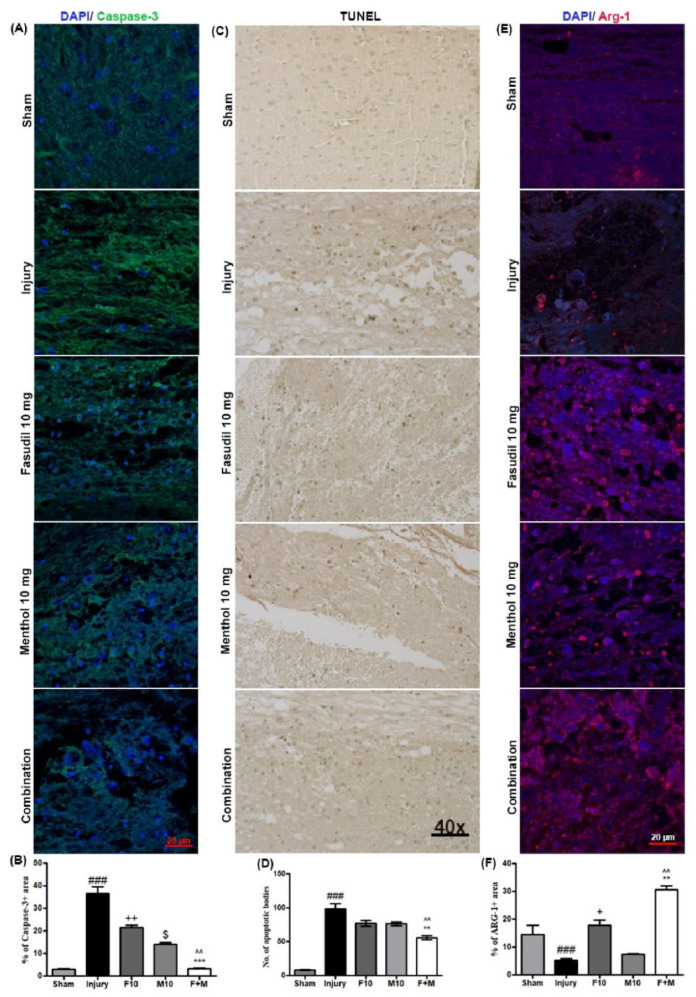
Combination of fasudil (5 mg) and menthol (10 mg) attenuates apoptosis and facilitates M2 polarization following spinal cord injury. (**A**) Representative immunofluorescence images for Caspase-3 and (**B**) quantitative fluorescence intensity of caspase-3. 4′,6-diamidino-2-phenylindole (DAPI) was used as an internal control (Blue). (**C**) Terminal deoxynucleotidyl transferase dUTP nick end labeling (TUNEL) assay showing apoptotic bodies following SCI in the representative spinal cord epicenter and (**D**) quantitative result of the TUNEL assay. (**E**) Representative immunofluorescence images of spinal cord epicenter for Arginase-1 (Arg-1) and (**F**) quantitative fluorescence intensity of Arginase-1. Scale bar: 20 µm, florescence quantification data are expressed in mean ± SEM, *n* = 3/group. F10, fasudil. 10 mg/kg i.p. M10, menthol 10 mg/kg i.p., F + M, Fasudil5 mg/kg + Menthol 10 mg/kg i.p., ### *p* < 0.001 (sham vs. injury), + *p* < 0.05, ++ *p* < 0.01 (fasudil vs. injury), $ *p* < 0.05 (menthol vs. injury), ^^ *p* < 0.05 (combination vs. fasudil 10 mg), and ** *p* < 0.01, *** *p* < 0.01 (combination vs. menthol 10 mg).

To compare the degree of apoptosis between the experimental groups, caspase-3 immunoreactivity was assessed in spinal cord samples at threedays post-SCI. The results of this comparison demonstrated that SCI-induced apoptotic cells remarkably increased in the injury-alone group (as determined by caspase-3 immunoreactivity) (Figure 4A) (### *p* < 0.001, sham vs. injury). However, a trend of marginal decrease in immunoreactivity was observed in the fasudil alone (++ *p* < 0.01 fasudil vs. injury), and menthol alone ($ *p* < 0.05 menthol vs. injury) treated groups. By contrast, a dramatic decrease in caspase-3 immunoreactivity was observed in the combined fasudil-treated and menthol-treated group spinal cords (Figure 4A,B) (^^ *p* < 0.01, combination vs. fasudil) (*** *p* < 0.001, combination vs. menthol). Furthermore, terminal deoxynucleotidyl transferase dUTP nick end labeling (TUNEL) assay, performed at three days post-SCI, revealed an increased number of apoptotic bodies in the injury alone group spinal cord samples (Figure 4C,D).

Expression of Arg-1, a marker of M2 macrophage, was also assessed by immunohistochemical staining at three days post-SCI. In the injury-only group, the expression of Arg-1 extensively decreased three days after SCI, which representsan inflammatory response. (Figure 4E) (### *p* < 0.001, sham vs. injury). The Arg-1 + area was increased after fasudil treatment (+ *p* < 0.05 fasudil vs. injury) or menthol treatment ($ *p* < 0.05 menthol vs. injury) compared to the injury group. In addition, a combination of fasudil and menthol promoted the immunomodulatory effect when compared to both fasudil-only (^^ *p* < 0.01, combination vs. fasudil) and menthol-only (*** *p* < 0.001, combination vs. menthol) treatment over the same period (Figure 4E,F).

### 3.4. Effects of Fasudil and Menthol Treatment on Glial and Fibrotic Scar Formation Following SCI

**Figure 5:**
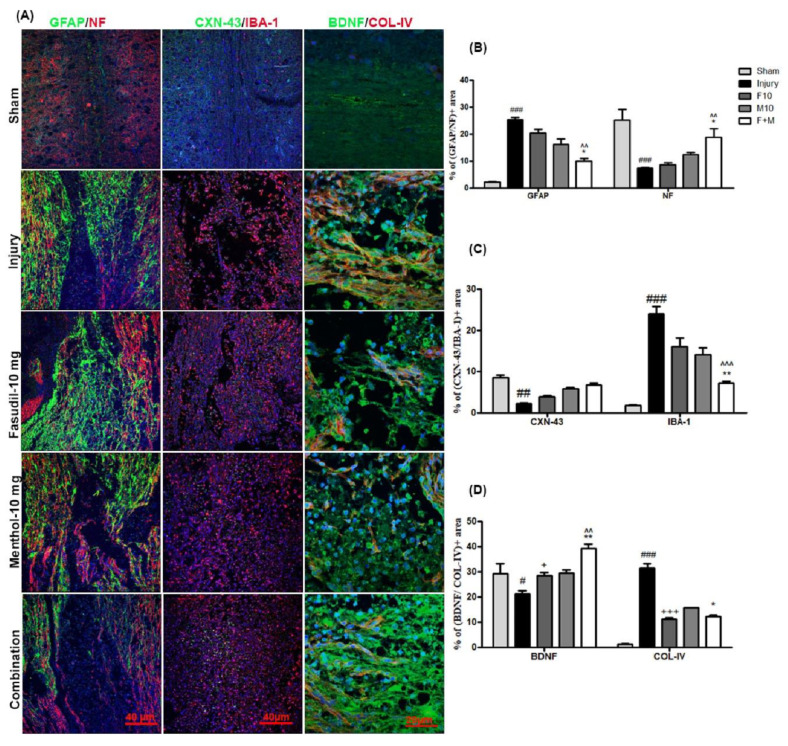
Combination of fasudil (5 mg) and menthol (10 mg) prevents glial scar formation and promotes neuroprotection following SCI. (**A**) Representative immunofluorescence images of spinal cord for glial fibrillary acidic protein (GFAP), neurofilament (NF), Connexin-43 (CXN-43), ionized calcium-binding adapter molecule -1 (IBA-1), brain-derived neurotrophic factor (BDNF), and collagen IV (Col-IV). (**B**) Quantitative fluorescence intensity of GFAP and NF, and (**C**) quantitative fluorescence intensity of CXN-43 and IBA-1, and (**D**) quantitative florescence intensity of BDNF and Col-IV (mean ± SEM, *n* = 2–3/group). Tissues were collected at 28 days after SCI. ### *p* < 0.001 (sham vs. injury), + *p* < 0.05, +++ *p* < 0.001 (fasudil vs. injury) ^^ *p* < 0.01, ^^^ *p* < 0.001 (combination vs. fasudilof 10 mg), and * *p* < 0.05, ** *p* < 0.01 (combination vs. menthol of 10 mg).

**Figure 6:**
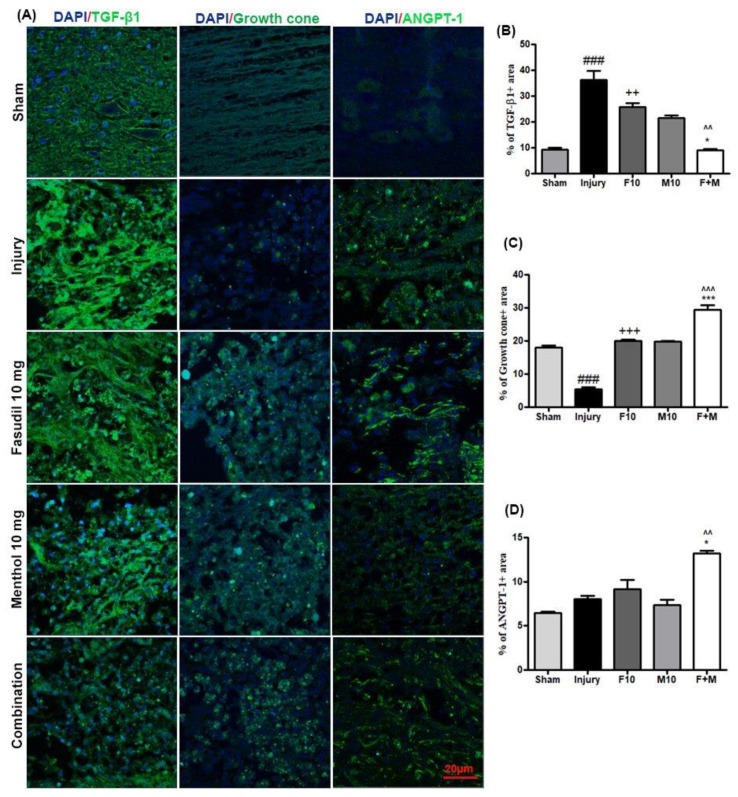
Combination of fasudil (5 mg) and menthol (10 mg) prevents fibrotic scarring and plays a significant role in angiogenesis and axonal regeneration following SCI. (**A**) Representative immunofluorescence images for transforming growth factor-β1 (TGF-β1), growth cone, and angiopoietin-1 (ANGPT-1). The experimental procedure is explained in the Materials and Methods section. (**B**) Quantitative fluorescence intensity of TGF-β1, (**C**) quantitative fluorescence intensity of growth cone, and (**D**) quantitative fluorescence intensity of ANGPT-1 (mean ± SEM, *n* = 2–3/group), (scale bar = 20 µm). Tissues were collected at 28 days after SCI. ### *p* < 0.001 (sham vs. injury), ++ *p* < 0.01, +++ *p* < 0.001 (fasudil vs. injury) ^^ *p* < 0.01, ^^^ *p* < 0.001 (combination vs. fasudil 10 mg), and * *p* < 0.05, *** *p* < 0.001 (combination vs. menthol 10 mg).

Glial scar formation at the epicenter of injured spinal cord is a widely-studied pathophysiological mechanism of the chronic phase after SCI. Astrocytes form the major portion of glial scars. Consequently, in the present study, the numbers of reactive astrocytes substantially increased in injury-alone spinal cords (as determined by GFAP immunoreactivity) (Figure 5A,B) (### *p* < 0.001, sham vs. injury). Similarly, substantially increased levels of microglial cells, which was determined by IBA-1 immunoreactivity, was induced after SCI in injury-alone spinal cord samples (Figure 5A,C) (### *p* < 0.001, sham vs. injury). In contrast, substantial inhibition of GFAP and IBA-1 expression was observed in combination therapy (^^ *p* < 0.01, ^^^ *p* < 0.001 combination vs. fasudil) (* *p* < 0.05, ** *p* < 0.01 combination vs. menthol). Prior reports have suggested that fibrotic glial scarring plays a significant role in hindering axonal growth. Coincidently, TGF-β1, which is a hallmark of fibrotic scarring, was extensively observed after SCI in the model used here (### *p* < 0.001, sham vs. injury), and was remarkably decreased after combined treatment with fasudil and menthol, whichsuggests optimal prevention of fibrotic scarring with this combined approach (Figure 6A,B) (++ *p* < 0.01, fasudil vs. injury) (^^ *p* < 0.01, combination vs. fasudil) (* *p* < 0.05, combination vs. menthol). The expression of collagen IV, which forms the dense basal lamina meshwork, was found to have exponentially increased at 28 days post-SCI in the injury-alone group (### *p* < 0.001, sham vs. injury). On the contrary, exponentially decreased collagen IV expression was observed in the combination group compared to single treatment with either drug alone (Figure 6A,D), which suggests considerably reduced fibrotic scarring (+++ *p* < 0.001, fasudil vs. injury) (* *p* < 0.05, combination vs. menthol).

### 3.5. Effects of Fasudil and Menthol Treatment on Angiogenesis and Neuroprotection

Immunoreactivity to the neurofilament (NF), which is a potent axonal marker, was significantly decreased in the injury-alone spinal cords and was significantly increased in the combined drug treatment group compared to the injury-alone group and single treatment with either drug alone (Figure 5A,B) (### *p* < 0.001, sham vs. injury) (^^ *p* < 0.01, combination vs. fasudil) (* *p* < 0.05, combination vs. menthol). Similarly, immunoreactivity to connexin-43 (CXN-43), which is a gap junctional marker, was significantly decreased following SCI (Figure 5A,C) (## *p* < 0.01, sham vs. injury) while increased immunoreactivity to CXN-43 was observed in the combination group compared to the individual fasudil-treated or menthol-treated spinal cord groups. In addition, the neuroprotective effects of combination therapy were evaluated by assessing the immunoreactivity of the BDNF. This revealed minimal BDNF expression in the injury-alone spinal cord samples (# *p* < 0.05, sham vs. injury)and significantly increased BDNF expression in the combined treatment group compared to the individual fasudil or menthol-treated spinal cord groups (Figure 5A,D) (^^ *p* < 0.01, combination vs. fasudil) (** *p* < 0.01, combination vs. menthol).

To further elucidate the endothelial protective and growth cone regenerative effects of combined treatment, we assessed the expression of the growth cone marker and ANGPT-1 at 28 days following SCI (Figure 6A). We found that growth cones were abundant in the sham spinal cord group and were dramatically decreased after injury (### *p* < 0.001, sham vs. injury), which then significantly increased in the fasudil and menthol-only treated groups (+++ *p* < 0.001, fasudil vs. injury). A significant increase in growth cone expression was also observed in the combined fasudil-and-menthol-treated group compared to the individual fasudil-treated or menthol-treated spinal cord groups (Figure 6A,C) (^^^ *p* < 0.001, combination vs. fasudil) (*** *p* < 0.001, combination vs. menthol).

ANGPT-1 expression was increased in the injury group relative to the sham group as well as in the fasudil-only group. ANGPT-1 expression was slightly decreased in the menthol-treated group. Contrastingly, significantly increased expression of ANGPT-1 was detected in the combination group compared to singular treatment with either drug alone, which reflects extensive angiogenesis (Figure 6A,D) (^ *p* < 0.05, combination vs. fasudil) (* *p* < 0.05, combination vs. menthol).

### 3.6. Effects of Fasudil and Menthol Treatment on Axonal Growth Following SCI

**Figure 7:**
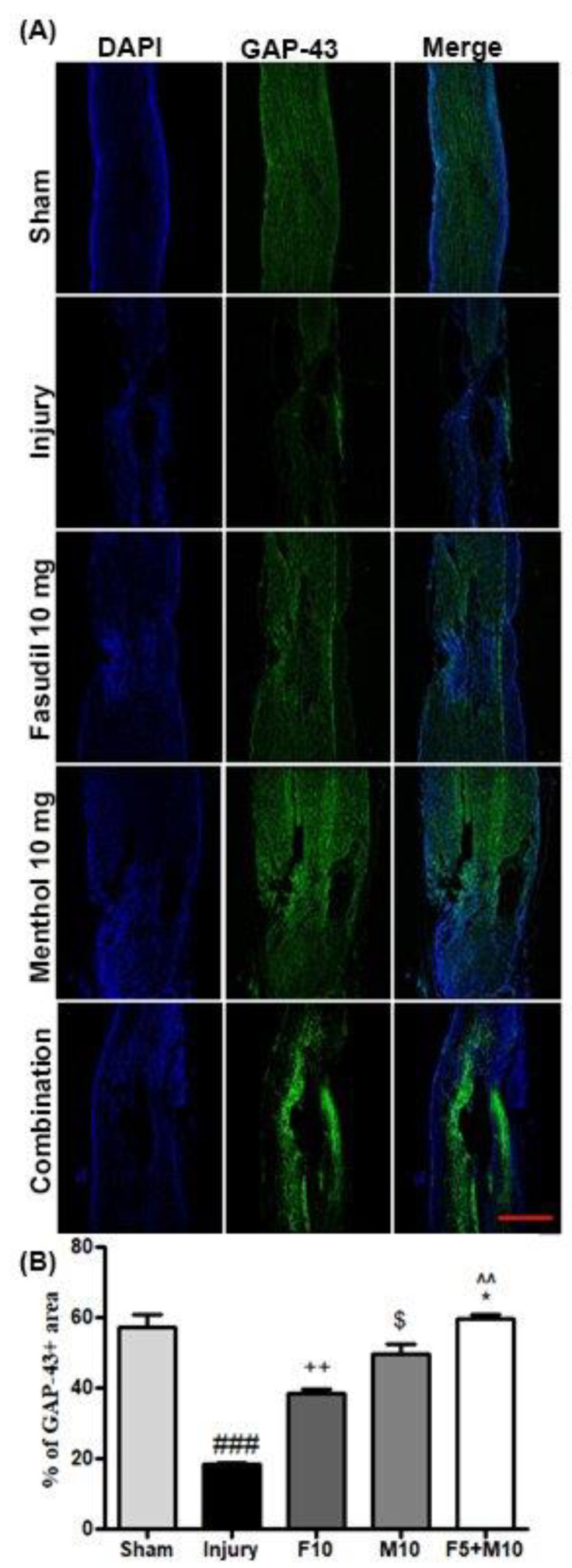
Combination of fasudil (5 mg) and menthol (10 mg) plays a significant role in growth cone formation following SCI. (scale bar = 750 µm) (**A**) Representative immunofluorescence images for growth-associated protein-43 (GAP-43) and (**B**) quantitative fluorescence intensity of Growth Associated protein-43 GAP-43. Data are expressed as mean ± SEM (*n* = 3/group) and analyzed by one-way ANOVA, which is followed by Neuman Keul’s post-test. Tissues were collected at 28 days after SCI. ### *p* < 0.001 (sham vs. injury), ++ *p* < 0.01 (fasudil 10 mg vs. injury), $ *p* < 0.05 (Menthol 10 mg vs. fasudil 10 mg) ^^ *p* < 0.01 (combination vs. fasudilof 10 mg), and * *p* < 0.05 (combination vs. menthol of 10 mg).

On the basis of our hypothesis, we expected that the prevention of glial and fibrotic scarring would enhance axonal growth following combination therapy. We, therefore, assessed growth-associated protein-43 (GAP-43) immunoreactivity at 28 days post-SCI. GAP-43 expression was decreased at 28 days post-SCI in the injury-alone group compared to that of the sham group (### *p* < 0.001, sham vs. injury) while single treatment with fasudil or menthol greatly prevented SCI-attenuated GAP-43 expression. Combined treatment led to significant increases in GAP-43 expression compared to the individual fasudil or menthol-treated spinal cord groups, which suggests optimal neuroprotection with this combined approach (Figure 7A,B) (^^ *p* < 0.01, combination vs. fasudil) (* *p* < 0.05, combination vs. menthol).

### 3.7. Effects of Fasudil and Menthol Treatment on Lesion Volume Following SCI

**Figure 8:**
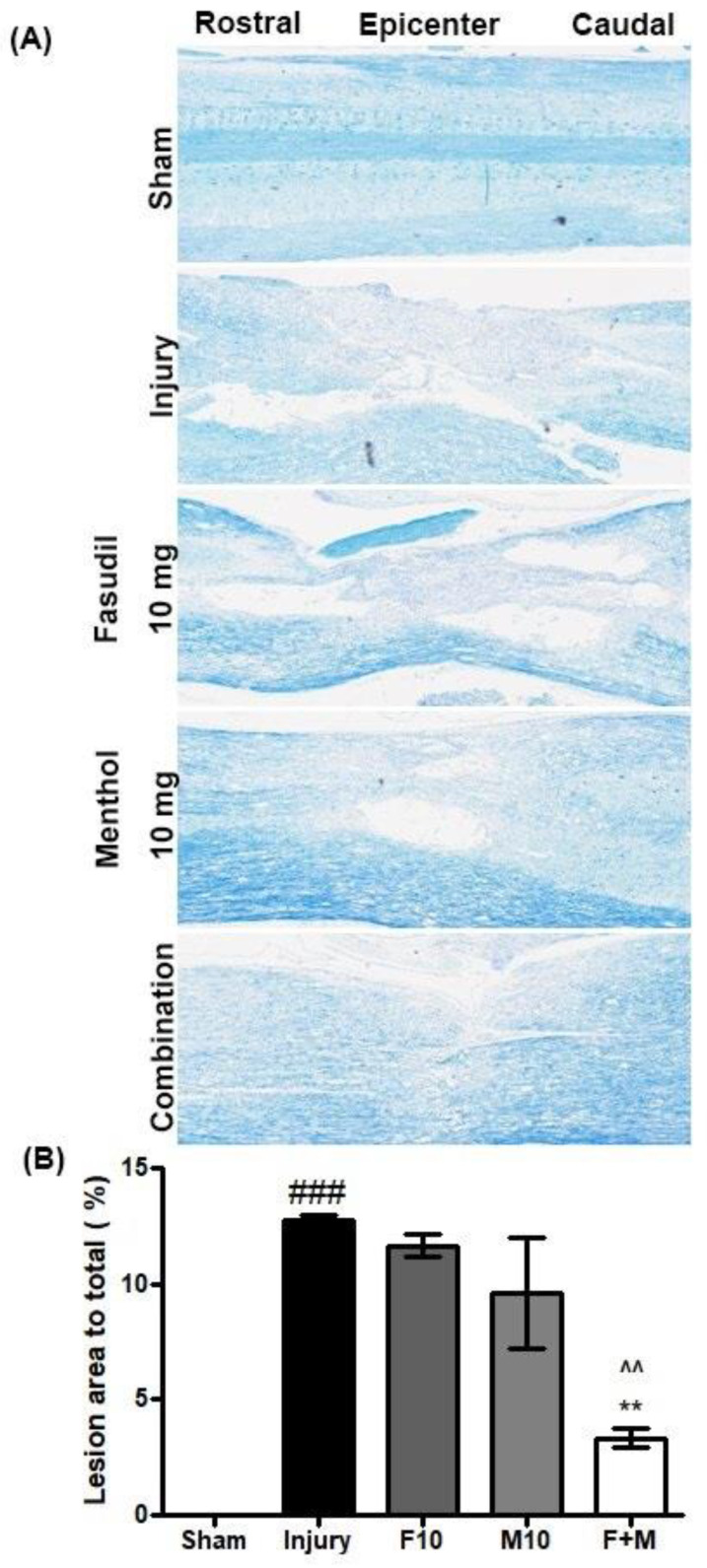
Combination of fasudil (5 mg) and menthol (10 mg) significantly diminishes the lesion area following SCI. (Lens = 10 x) (**A**) Representative images of spinal cords stained with Luxol fast blue (LFB) and (**B**) measurement and quantification of lesion area (mean ± SEM, *n* = 2–3/group). Tissues were collected at 28 days after SCI. Combination, Fasudil5 mg/kg + Menthol 10 mg/kg i.p., ### *p* < 0.05 (sham vs. injury), ^^ *p* < 0.01 (combination vs. fasudilof 10 mg), and ** *p* < 0.01 (combination vs. menthol of 10 mg).

Luxol fast blue (LFB) staining was performed and the lesion area was measured at 28 days post-SCI. As shown in Figure 8A,B, representative spinal cords from injury-alonerats had a significantly larger lesion area (percentage lesion area: lesion area to total spinal cord area, 12.73 ± 0.25%) than the sham (### *p* < 0.001, sham vs. injury) anda larger area than individual fasudil-treated and menthol-treated spinal cords samples with average lesion areas of 11.6584 ± 0.49% and 9.59 ± 2.37%, respectively. Spinal cord samples from the fasudil-only or menthol-only treatment groups exhibited inconsiderable decrements in the lesion area compared to the injury-only group. By contrast, substantial lesion area reductions were observed in the combined fasudil-treated and menthol-treated group spinal cord samples, which had a mean lesion area of 3.33 ± 0.42% when compared to individual fasudil-treated and menthol-treated groups (Figure 8B) (^^ *p* < 0.01, combination vs. fasudil) (*** *p* < 0.001, combination vs. menthol).

## 4. Discussion

Recovery from SCI is a challenging process due to a lack of effective treatment options for repairing neural damage and alleviating functional impairments. In this study, we devised a drug combination method to enhance the therapeutic effect of fasudil. While inhibiting the RhoA-ROCK signaling pathway plays a critical role in neurite regeneration, regulation of Ca^2+^ homeostasis also occupies an important position in programmed cell death. For example, various proteolytic enzymes including caspase were activated when triggered by increased cytosolic Ca^2+^ [30]. Therefore, we hypothesized that combination of fasudil and other neuronal calcium current modulators would promote rehabilitation after SCI. We established three conditions to look for drug candidates that could be combined with fasudil. First, to administrate safely, the drug had to be approved as safe by the constitutions. Second, it had to be able to function as a modulator to block calcium influx and inflammation, and it also had to be proven to act on central nervoussystem (CNS). Menthol is an FDA-approved generally recognized as safe material that satisfies all three of the above conditions. Sidell et al. reported that menthol blocks dihydropyridine insensitive Ca^2+^ channels and induces neurite outgrowth in neuroblastoma cells [24]. Ton et.al. reported that administration of menthol inhibited acetylcholine-induced or nicotine-stimulated calcium influx into neurons via an allosteric mechanism [31].

The in vitro experiment was conducted to evaluate drug effectiveness and concordance between previous studies. Lipopolysaccharides (LPS), which was known as inflammatory stimuli for N2a cells [30], was used as experimental reproduction of the SCI condition. While fasudil promotes neurite outgrowth in vitro, when treated in combination with LPS, the cell viability of N2a cells were declined. This result suggests that the effect of fasudil on neurite outgrowth would be reduced when treated in an acute injury condition. However, when LPS was treated in combination with fasudil and menthol, the percentage of neurite outgrowth and cell viability of N2a cells were significantly increased when compared with LPS with the fasudil treatment group, which led to the hypothesis that combination of fasudil and menthol would increase the effect of fasudil on neurite outgrowth in the SCI condition.

The in vivo experiment results illustrated motor function impairment and neuropathic pain sensation after SCI. Earlier studies showed that the fasudil promoted functional recovery following SCI [14,15]. Herein, a combination of fasudil and menthol showed faster recovery in motor function, such as joint movements, stepping, or forelimb-hindlimb coordination, and faster recovery from neuropathic pain when compared to either drug treatmentalone. Furthermore, caspase-3 is an important marker of apoptosis.It also contributes to the rho-independent activation of ROCK kinase [11]. In addition, the earlier research suggested that menthol inhibits the apoptotic response after SCI and may, thus, reduce ROCK activity, which contributes to neuroprotection after SCI [20]. In our results, SCI-induced apoptotic bodies were significantly decreased after three days of combined treatment. In addition, the immunoreactivity of Arg-1, which is the potent M2 marker, was extensively decreased threedays after SCI and exponentially increased with combination therapy, as compared to both fasudil-only and menthol-only treatment over the same period. Even the amount of Arg-1 expression was not significantly improved in the menthol-only treatment group.This group exhibited significant decreases in caspase-3 relative to the injury or fasudil-only treatment groups.

We further found that, in the injury group, considerable amounts of Glial fibrillary acidic protein (GFAP), a glial scar marker, ionized calcium-binding adaptor molecule-1(IBA-1), a microglial marker that indicates neuroinflammation after SCI [31], and collagen-IV were expressed after SCI. Since the glial scars interfere with neuronal regeneration of neurons and block nerve conduction [32], inhibition of scar formation is essential in order to prevent permanent disability in patients with spinal cord injury. By contrast, GFAP expression was decreased in the tissue samples treated with a combination of fasudil and menthol. While some neuroprotective benefits of glial scars, which seal injured tissues and prevent damage spread [33], have been suggested, we found a negative correlation between GFAP expression and neurofilament expression in the present study. GFAP expression was also negatively correlated with BDNF, which is a potent neurotrophic factor, as well as functional recovery, represented by the BBB score. BDNF has neuroprotective effects on varieties of neuronal populations following SCI. Research also shows that BDNF prevents glutamate-induced apoptotic cell death [34]. Similarly, the neurofilament, which is a cytoskeleton protein abundantly expressed in larger axons, was considerably diminished in the SCI injured, untreated spinal cord. However, there was an exponentially increased number of filamentous proteins in the combined fasudil-and-menthol-treated spinal cord samples. In terms of glial scar formation, remarkable decreases in GFAP, IBA-1, and collagen-IV were also observed the spinal cord samples from the combined therapy group compared to those from the injury-alone group. In addition, a decreased amount of CXN-43, which represents junctional destruction, occur after SCI [35], while greatly increased expression of CXN-43 was noted in this scenario in the combination therapy group, illustrating the stabilization of gap junctions.

Additionally, immunoreactivity of TGF-β1 (marker for fibrotic scar) shows that, with substantial TGF-β1 expression, extensive fibrotic scarring was experienced in injured and untreated rats. However, in the combination group, scarring was significantly diminished. Angiopoietins (ANGPTs) are vascular growth factors, which attribute to the blood vessel formation and maturation [25]. ANGPT-1 is a potent angiogenic regulator, which prevents inflammation and vascular leakage and is expressed in the number of cells especially in smooth muscle cells and pericytes [36]. Herein, fluctuated ANGPT-1 expression was experienced between the experimental groups. Neo-vasculatures were observed marginally more in the injury group compared to the sham and marginal higher expression in treatment, especially a significant number of micro-vessels in the combination therapy-treated spinal cord.

In addition, GAP-43 is associated with nerve regeneration, nerve sprouting, and long-term axonal potentiation [37]. In this study, we examined the immunoreactivity of GAP-43 and growth cones with existing findings demonstrating that extensive neurodegeneration occurred in spinal cords after SCI. Spinal cord of injury-only group mice showed the lowest levels of GAP-43 expression and growth cone formation. On the contrary, significant GAP-43 expression and growth cone formation occurred with combined therapy relative to individual drug-treated spinal cords.

Menthol was found to have analgesic effects [21]. In the present study, we found that neurological outcomes in the menthol-treated SCI group indicated significantly diminished neuropathic pain. With profound pain recovery, the presented effect of menthol was reflected in a combinationof menthol and fasudil-treated group. Rates of functional recovery are often based on re-myelination and lesion area decrements [38]. In the present study, compared to the remaining groups, substantial decreases in lesion area occurred in the combination-treated group. Despite the combination group receiving less fasudil (5 mg/kg) than the fasudil-only treated group (10 mg/kg), it was in the combination group that functional recovery was most markedly restored.

The combination ROCK inhibitor fasudil and celecoxib enhanced the functional recovery after spinal cord injury in rats via the synergistic mechanism [18]. However, thisstudy had some limitations such as the drugs were treated from two different routes (celecoxib (orally) and fasudil (intramuscularly)). Ideally, both drugs should be delivered from the same route. Additionally, celecoxib is a prescription nonsteroidal anti-inflammatory drug (NSAID), which is associated with serious side effects such as: gastrointestinal bleeding, diarrhea, gastroesophageal reflux disease, and serious liver problems. Similarly, research illustrates that combined therapy of bone marrow stromal cells and ROCK inhibitor fasudil enhances axonal regeneration after SCI [39]. In this study, fasudil was also administered through the intraperitoneal route, whereas bone marrow stromal cells were directly transplanted into the spinal cord. Therefore, they also did not treat the drug through the same route. Moreover, stem cell transplantation is associated with an immune reactionin some cases. These shortcomings are limited due to acombination with menthol.

Nevertheless, in this study, there are still limitations to be overcome. Despite the combined fasudil and menthol showing promising neuroprotective pharmacological effects, the exact pathophysiological and pharmacological mechanism of synergism remains to be elucidated. Further study is also needed for the systemic effect of the combined drug treatment and elucidation of the exact mechanism governing the synergic effect between fasudil and menthol. Analyzing calcium influx, which participates in the process of apoptosis after injury and which is modulated by menthol [24] is also needed. In addition, since fasudil is already undergoing clinical trial and menthol is approved by FDA as safe food additives, a systemic side effect of drug combination is unlikely to appear.

Therefore, in thefuture, we will focus on finding out the exact mechanism of the drug by investigating the effects of combined therapy in other associated disorders like neuropathic pain, multiple sclerosis, cerebral infarction, etc. Additionally, we would like to investigate the possibility of delivering the drug in a better route of administration such as targeted drug delivery. Essentially, existing data demonstrates that the combination of fasudil and menthol could be a potential therapeutic strategy for restoring an injured spinal cord.

## 5. Conclusions

In conclusion, combined treatment of fasudil and menthol improve functional recovery by alleviating apoptosis, inflammation, and glial scar formation and by promoting neovascularization and remarkable neuroprotection. These results suggest that combined treatment of fasudil and menthol may be a promising strategy to treat SCI.

## Abbreviations

ANGPT-1	Angiopoietin-1
BBB	Basso, Beattie, and Bresnahan
BDNF	Brain-derived neurotrophic factor
Col-IV	Collagen IV
CXN-43	Connexin-43
COX-2	Cyclooxygenase-2
GFAP	Glial fibrillary acidic protein
GAP-43	Growth-associated protein-43
i.p.	Intraperitoneally
IBA-1	Ionized calcium-binding adapter molecule -1
LPS	Lipopolysaccharide
LFB	Luxol fast blue
N2a	Neuro2A
NF	Neurofilament
O.D.	Optical density
PFA	Paraformaldehyde
PWT	Paw withdrawal threshold
RhoA	Ras Homolog Family Member A
ROCK	Rho-associated kinase
SCI	Spinal cord injury
SEM	standard error of the mean
TUNEL	Terminal deoxynucleotidyl transferase dUTP nick end labeling
